# Serum Cardiac Troponin-I is Superior to Troponin-T as a Marker for Left Ventricular Dysfunction in Clinically Stable Patients with End-Stage Renal Disease

**DOI:** 10.1371/journal.pone.0134245

**Published:** 2015-08-03

**Authors:** Maurits S. Buiten, Mihály K. de Bie, Joris I. Rotmans, Friedo W. Dekker, Marjolijn van Buren, Ton J. Rabelink, Christa M. Cobbaert, Martin J. Schalij, Arnoud van der Laarse, J. Wouter Jukema

**Affiliations:** 1 Department of Cardiology, Leiden University Medical Center (LUMC), Leiden, The Netherlands; 2 Department of Nephrology, LUMC, Leiden, The Netherlands; 3 Department of Clinical Epidemiology, LUMC, Leiden, The Netherlands; 4 Department of Nephrology, HAGA, The Hague, The Netherlands; 5 Department of Clinical Chemistry and Laboratory Medicine, LUMC, Leiden, The Netherlands; Fondazione G. Monasterio, ITALY

## Abstract

**Background:**

Serum troponin assays, widely used to detect acute cardiac ischemia, might be useful biomarkers to detect chronic cardiovascular disease (CVD). Cardiac-specific troponin-I (cTnI) and troponin-T (cTnT) generally detect myocardial necrosis equally well. In dialysis patients however, serum cTnT levels are often elevated, unlike cTnI levels. The present study aims to elucidate the associations of cTnI and cTnT with CVD in clinically stable dialysis patients.

**Methods:**

Troponin levels were measured using 5^th^ generation hs-cTnT assays (Roche) and STAT hs-cTnI assays (Abbott) in a cohort of dialysis patients. Serum troponin levels were divided into tertiles with the lowest tertile as a reference value. Serum troponins were associated with indicators of CVD such as left ventricular mass index (LVMI), left ventricular ejection fraction (LVEF) and the presence of coronary artery disease (CAD). Associations were explored using regression analysis.

**Results:**

We included 154 consecutive patients, 68±7 years old, 77% male, 70% hemodialysis. Median serum cTnT was 51ng/L (exceeding the 99^th^ percentile of the healthy population in 98%) and median serum cTnI was 13ng/L (elevated in 20%). A high cTnI (T3) was significantly associated with a higher LVMI (Beta 31.60; p=0.001) and LVEF (Beta -4.78; p=0.005) after adjusting for confounders whereas a high serum cTnT was not. CAD was significantly associated with a high cTnT (OR 4.70 p=0.02) but not with a high cTnI. Unlike cTnI, cTnT was associated with residual renal function (Beta:-0.09; p=0.006).

**Conclusion:**

In the present cohort, serum cTnI levels showed a stronger association with LVMI and LVEF than cTnT. However, cTnT was significantly associated with CAD and residual renal function, unlike cTnI. Therefore, cTnI seems to be superior to cTnT as a marker of left ventricular dysfunction in asymptomatic dialysis patients, while cTnT might be better suited to detect CAD in these patients.

## Introduction

In patients with end-stage renal disease (ESRD) cardiovascular disease (CVD) is the most important cause of death, accounting for approximately 40% of mortality [1]. Accordingly, accessible biomarkers for the identification and quantification of CVD in these patients are crucial. In the general population, cardiac troponin levels are widely used for the detection of myocardial injury [2]. Several studies have shown that cardiac troponins can predict cardiovascular and all-cause mortality in clinically stable patients with ESRD as well [3–6]. Therefore, serum cardiac troponin might be a valuable biomarker to identify the presence and severity of CVD in patients with ESRD.

Troponins control cardiac muscle contraction by facilitating calcium-mediated actin and myosin interaction in cardiomyocytes. The cardiac-specific isoforms of troponin-I (cTnI) and troponin-T (cTnT) have a comparable sensitivity and specificity for the detection of myocardial injury in the general population [7]. In dialysis patients, serum cTnT levels exceed the 99^th^ percentile of a healthy population in most cases, whereas cTnI is only elevated in 15–30% [8–10]. Furthermore, it has been shown that circulating cTnT undergoes fragmentation, facilitating renal clearance, whereas cTnI might be predominantly cleared by other pathways such as the liver [11–14]. In view of these differences, the question arises whether cardiac troponins are useful in the detection of CVD in dialysis patients and if so, whether both cardiac-specific isoforms are comparable in their ability to detect CVD.

Data that compare the association of both cardiac-specific troponins with CVD in ESRD is limited, especially using high sensitive assays [5, 8, 10]. The aim of the present study was to evaluate the association of both cTnI and cTnT with the presence of CVD in a cohort of clinically stable ESRD patients on dialysis therapy, using state of the art high-sensitivity troponin assays.

## Materials and Methods

### Population and design

For this study all participants currently included in the prospective ICD-2 trial (ISRCTN20479861) were analyzed [15]. In short, the ICD-2 trial is an ongoing randomized controlled clinical trial designed to evaluate the effectiveness of an implantable cardioverter defibrillator (ICD) in the prevention of sudden cardiac death in dialysis patients. Patients are randomized for an ICD or no ICD. The study protocol has been described previously [15]. All participants are between 55 and 80 years old, treated with either hemodialysis (HD, typically 3 times a week) or peritoneal dialysis (PD) and have a left ventricular ejection fraction (LVEF) ≥35%. Patients with an acute myocardial infarction (AMI) in the last 40 days were excluded. All patients provided written informed consent and the trial was approved by the local medical ethics committee (Medisch Ethische Commissie, LUMC, Leiden, The Netherlands).

All included patients underwent extensive screening at the time of enrollment in the ICD-2 trial, including blood analysis, computed cardiac tomography angiography (CTA) and transthoracic echocardiography (TTE). Blood was collected on a non-dialysis day for patients on HD and between dialysis sessions for patients on PD. Furthermore, data on demographic characteristics, coexisting conditions and information regarding the dialysis procedures were collected. Residual renal function (RRF) was calculated as the mean of creatinine and urea clearance in a 24-hour urine sample adjusted for body surface area (mL/min per 1.73m^2^) [16]. The means of both post- and pre-dialysis plasma samples were used to estimate mean plasma creatinine and urea concentrations [16]. RRF was considered zero in patients with a urinary output <100 mL/24h.

### Laboratory tests

The blood samples were centrifuged after collection at baseline and serum high-sensitivity cTnT (hs-cTnT) concentration was assayed using the Elecsys Troponin-T 5^th^ generation high-sensitivity assay (Roche Diagnostics, Penzberg, Germany), with a limit of detection of 5 ng/L and a 99^th^ percentile in the healthy population of 14.0 ng/L. The manufacturer recommends a cut-off for a positive test (for myocardialinjury) of 14.0 ng/L. Additional blood samples were separated in multiple vials per sample and stored at -80°C for future assays. In patients of whom the 5^th^ generation hs-cTnT assay was not available at time of inclusion, serum hs-cTnT level was measured from the frozen samples. High-sensitivity cTnI was assayed from frozen samples of all patients using the Architect STAT High-Sensitivity Troponin-I assay (Abbott Laboratories, Abbott Park, Illinois, US) with a limit of detection of 1.2 ng/L and a 99^th^ percentile in the healthy population of 26.2 ng/L [17]. The manufacturer recommends a cut-off for a positive test of 26.2 ng/L.

### Multi Slice CT protocol

The CTA protocol used has been previously described [18]. Patients were scanned using a 64-slice CT scanner (Aquillion64, Toshiba Medical Systems, Otawara, Japan) or a 320-slice CT scanner (Aquilion ONE, Toshiba Medical Systems). Observers interpreted the studies, while blinded for laboratory results. Post- and pre-hydration were performed in accordance with the nephrologist. Coronary artery disease (CAD) was deemed present in case of ≥50% luminal narrowing in at least one coronary artery or if coronary artery bypass grafting (CABG) or a percutaneous coronary intervention (PCI) was performed in the past.

### Echocardiography

All patients underwent 2-dimensional TTE with commercially available ultrasound equipment (M3s probe, Vivid 7, GE Vingmed, Horton, Norway). The images were digitally stored for off-line analysis (EchoPAC version 110.0.0, GE Vingmed). Observers interpreted the studies, while blinded for laboratory results. LVEF was computed using Simpson’s biplane method and LV mass index (LVMI) was calculated with the modified American Society of Echocardiography equation indexed for body surface area [19].

### Statistical analysis

Baseline characteristics were presented as mean ± standard deviation or as median with interquartile range (IQR). Dichotomous data were presented as proportions. Univariate linear regression analysis was performed to assess the association between serum cTnT and cTnI levels and baseline characteristics. Serum cTnT and cTnI levels were log-transformed to acquire a normal distribution for the univariate regression analyses.

The association between serum troponin levels and LVMI or LVEF was assessed using linear regression models with unstandardized Betas. For the association between serum troponin levels and the presence of CAD, logistic regression was used with odds ratios (OR). Serum cTnT and cTnI levels were divided into tertiles (T1–T3) and implemented as ordinal values in the various regression models. This was done to facilitate interpretation of Betas and ORs and to allow comparison of cTnT and cTnI effects. Tertile 1(T1) was used as a reference.

Model 1 assessed the crude association with cTnT and cTnI, model 2 was adjusted for age and gender, model 3 was additionally adjusted for diabetes and hypertension, and in model 4 additional adjustments for dialysis type, dialysis vintage and RRF were made.

All statistical analyses were performed using SPSS (version 20.0, IBM Corp., Amonk, NY, USA). All statistical tests were two-sided and a p-value <0.05 was considered statistically significant.

## Results

### Baseline characteristics

A total of 154 consecutive, clinically stable dialysis patients were included in the present study (S1 Dataset). The average age of patients was 68±7 years, 77% was male and 70% utilized HD as modality for renal replacement therapy, while the other 30% performed PD. Baseline characteristics are shown in Table 1. The primary causes of renal failure were: hypertension (n = 38; 25%), diabetes mellitus (n = 34; 22%), glomerulonephritis (n = 19; 12%), acute tubular necrosis (n = 6; 4%), polycystic disease (n = 5; 3%), malignancy (n = 4; 3%), other (n = 28; 18%), or unknown (n = 20; 13%).

**Table 1:** Baseline Characteristics.

Total cohort	n = 154
Age, years	68±7
Male gender	118 (77%)
Systolic BP, mmHg	138±22
Diastolic BP, mmHg	75±10
BMI, kg/m^2^	27±5
History of smoking	96 (62%)
Hypertension	125 (81%)
Diabetes	54 (35%)
Hypercholesterolemia	73 (47%)
Dialysis vintage, years	1.5 (0.8–2.5)*
RRF, ml/min/1.73m^2^	1.8 (0–2.7)*
Dialysis modality, HD	109 (71%)
cTnT, ng/L	51.0 (34.8–75.3)*
cTnI, ng/L	13.1 (7.8–23.0)*
LVMI, g/m^2^	128±40
LVEF, %	53±7
Presence of CAD	89 (58%)

Indicated are numbers (percentages), means ± SD, and *medians (IQR). BP: Blood Pressure, BMI: Body Mass Index; RRF: Residual Renal Function; HD: Hemodialysis; cTnT: cardiac Troponin-T; cTnI: cardiac Troponin-I; LVMI: Left Ventricular Mass Index; LVEF: Left Ventricular Ejection Fraction; CAD: Coronary Artery Disease.

**Fig 1:**
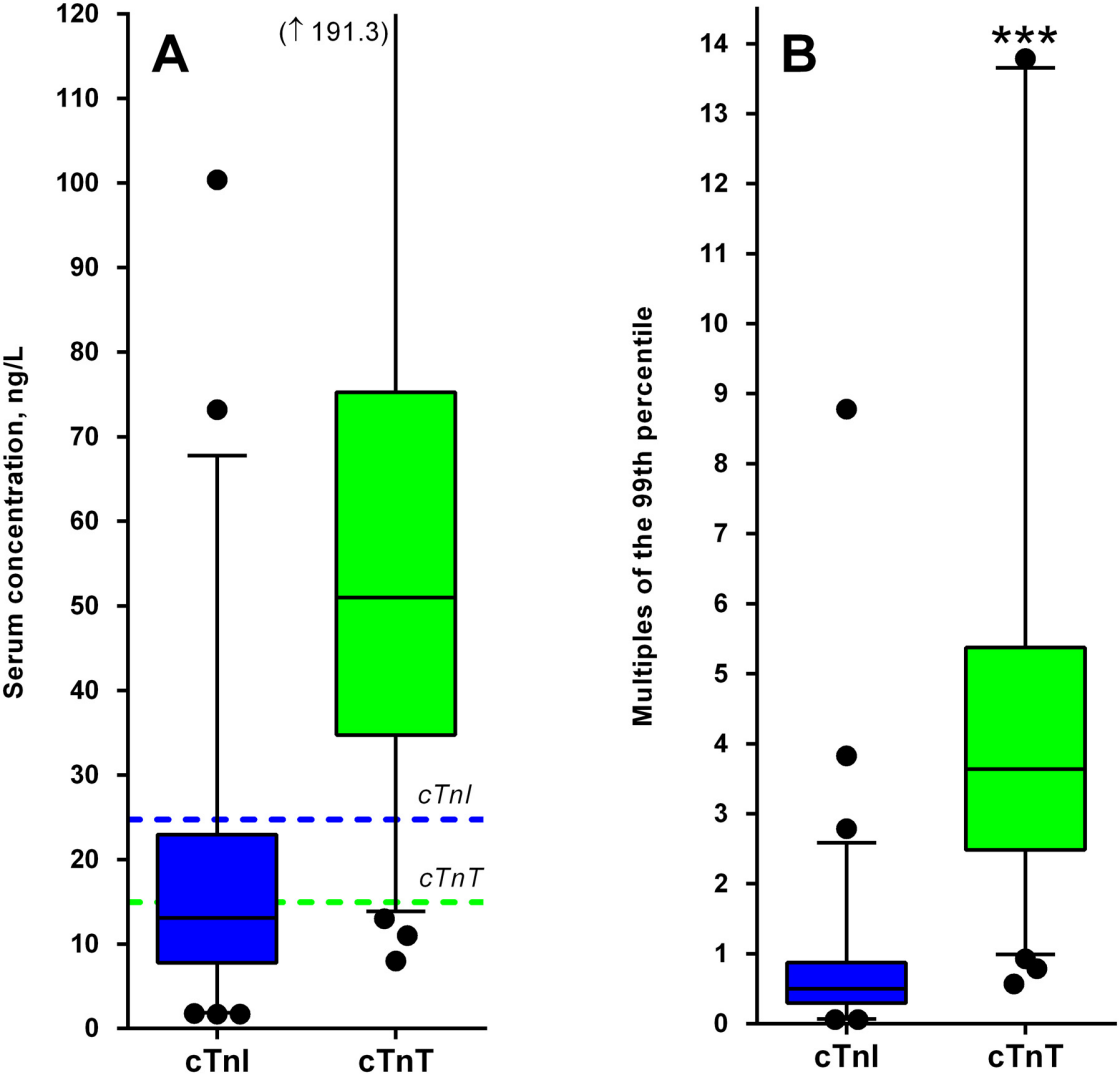
Distribution of serum cTnI and serum cTnT. (A) Box Whisker plot of serum cTnI and cTnT concentrations indicating the median, interquartile range, 2.5^th^ and 97.5^th^ percentile. The dotted lines represent the 99^th^ percentile cut-off for a positive test. (B) Box and Whisker plot of serum cTnI and cTnT expressed as multiples of the 99^th^ percentile. ***; p<0.001.

Median serum cTnT was 51.0 ng/L (IQR: 34.8–75.3 ng/L) and median serum cTnI was 13.1 ng/L (IQR: 7.8–23.0 ng/L). A total of 151 patients (98%) had a cTnT level above 14 ng/L, the predefined cut-off for a positive test. On the other hand, the cTnI test was positive (>26.2 ng/L) in only 30 patients (20%). Calculated as multiples of the 99^th^ percentile, serum cTnT was 3.6-fold increased (IQR 2.5–5.4 ng/L) compared to 0.7-fold increase in cTnI (IQR 0.3–0.9 ng/L), which was significantly different (p<0.0001). The distributions of cTnT and cTnI are shown in Fig 1. cTnT showed a positive association with cTnI (Beta:0.95; r = 0.72; p<0.001).

Serum samples were stored for a period of 0.8–82.0 months before serum cTnI measurement. The mean cTnI value in the quartile of samples with the longest storage time, compared to those with the shortest storage time, did not differ significantly (22.9 versus 17.0 ng/L, p = 0.38). The mean serum cTnT levels measured from the frozen samples were comparable to the cTnT levels from the samples measured immediately at baseline (64.5 versus 61.7 ng/L, p = 0.7).

Univariate associations between both troponin isoforms and baseline characteristics are shown in Table 2. Serum cTnT was positively correlated with age (Beta:0.03 per year increase; p<0.001), gender (Beta:0.41 for males; p = 0.001), hypertension (Beta:0.35; p = 0.01), dialysis vintage (Beta:0.03 per year increase; p = 0.02), RRF (Beta:-0.09 per unit increase; p = 0.01), and dialysis modality (Beta:-0.27 for HD; p = 0.02). Serum cTnI showed results generally comparable with cTnT with a positive association with age (Beta:0.03; p<0.001), gender (Beta:0.62 for males; p<0.001), hypertension (Beta:0.73; p<0.001) and dialysis vintage (Beta:0.04; p = 0.04). In contrast to cTnT, cTnI was not associated with dialysis modality or RRF.

**Table 2:** Univariate association of serum troponins with baseline characteristics using linear regression.

	Troponin-T, ng/L		Troponin-I, ng/L	
	Beta	p-value	Beta	p-value
Age, years	0.03	<0.001	0.03	<0.001
Male gender	0.41	0.001	0.62	<0.001
Systolic BP, mmHg	0.002	0.43	0.002	0.46
Diastolic BP, mmHg	-0.002	0.71	-0.01	0.49
BMI, kg/m^2^	0.02	0.15	0.02	0.29
History of smoking	0.11	0.30	0.23	0.13
Hypertension	0.35	0.01	0.73	<0.001
Diabetes	0.14	0.18	0.20	0.18
Hypercholesterolemia	-0.09	0.40	-0.04	0.79
Dialysis vintage, years	0.03	0.02	0.04	0.04
RRF, ml/min/1.73m^2^	-0.09	0.01	-0.02	0.74
Dialysis modality, HD	-0.27	0.02	-0.17	0.27

Serum cTnT and cTnI levels were log transformed to acquire a normal distribution. BP: Blood Pressure, BMI: Body Mass Index; RRF: Residual Renal Function; HD: Hemodialysis.

### Cardiovascular disease

**Fig 2:**
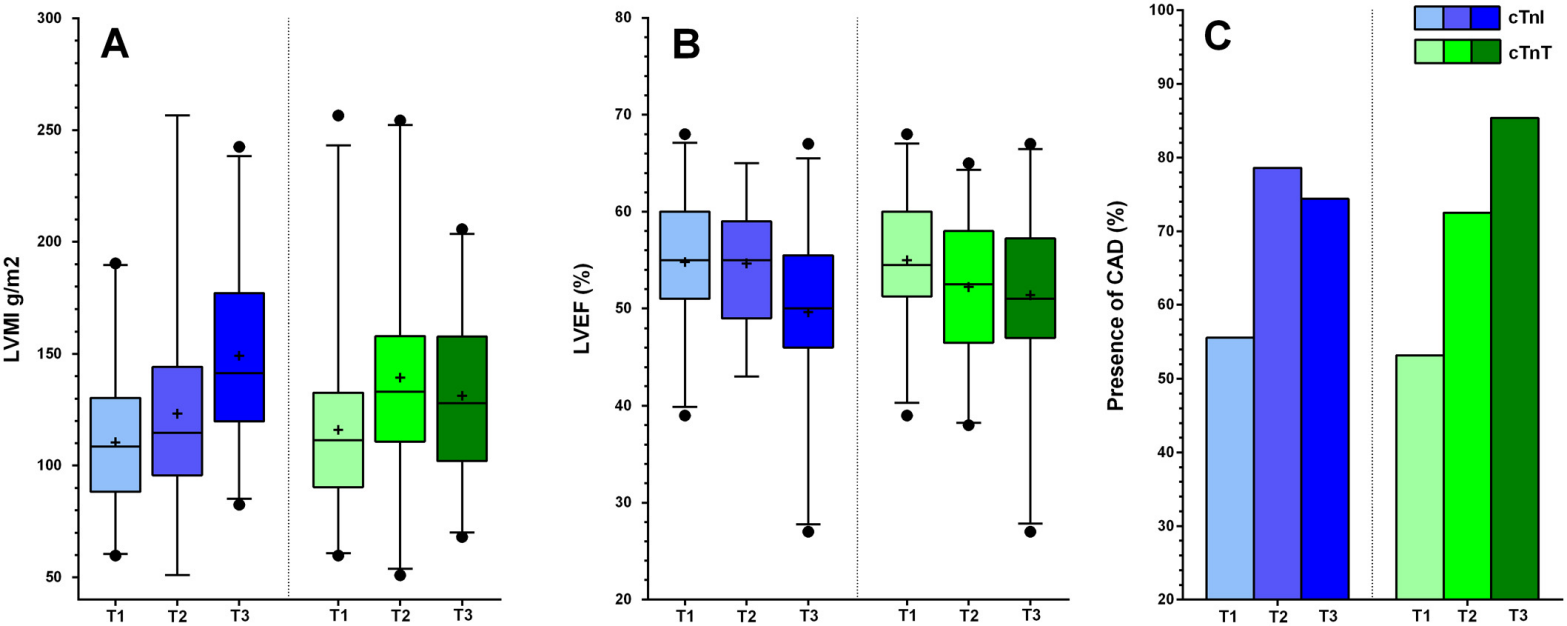
Unadjusted markers for cardiovascular disease divided per tertile of cTnI and cTnT. Box and Whisker plot of serum cTnI and cTnT concentrations divided into tertiles (T1, T2, T3) indicating the median, interquartile range, 2.5^th^ and 97.5^th^ percentile for unadjusted LVMI (A) and LVEF (B). The percentage of patients with significant CAD divided per tertile of serum cTnI and cTnT are shown in graph C. Serum cTnI is shown in shades of blue and serum cTnT is shown in shades of green. LVMI: Left Ventricular Mass Index; LVEF: Left Ventricular Ejection Fraction; CAD: Coronary Artery Disease.

Several multivariate models were built to assess the association between different tertiles of both troponin isoforms and our markers of CVD. The distribution of both troponins over the different tertiles were: cTnT; T1:8.0–40.0 ng/L, T2:41.0–64.0 ng/L, T3:65.0–379.0 ng/L cTnI; T1 1.7–9.4 ng/L, T2:9.6–17.5 ng/L, T3:17.9–230.0 ng/L. In model 1, the crude association between troponin and a specific condition of CVD was tested. Model 2 was adjusted for age and gender. Model 3 was adjusted for risk factors of CVD such as diabetes and hypertension, on top of the variables included in model 2. Finally, in model 4 we adjusted for factors associated with ESRD, such as dialysis type (HD or PD), dialysis vintage and RRF, as well as all variables previously mentioned. All multivariate models are shown in Tables 3 and 4. Fig 2 shows the distribution of LVMI, LVEF and the presence of CAD in the three tertiles of cTnI and cTnT.

**Table 3:** Linear regression for the association between tertiles of serum cardiac troponins, LVMI and LVEF.

**LVMI**	**cTnI**	**Beta**	**p-value**	**cTnT**	**Beta**	**p-value**
Model 1. Crude association	T1 vs. T2	12.78	0.09	T1 vs. T2	23.42	0.004
	T1 vs. T3	38.72	<0.001	T1 vs. T3	15.28	0.05
Model 2. + Age & Gender	T1 vs. T2	10.25	0.19	T1 vs. T2	18.23	0.03
	T1 vs. T3	33.53	<0.001	T1 vs. T3	9.51	0.23
Model 3. + Diabetes & Ht	T1 vs. T2	9.02	0.28	T1 vs. T2	16.51	0.05
	T1 vs. T3	32.42	<0.001	T1 vs. T3	8.00	0.33
Model 4. + Dialysis factors	T1 vs. T2	9.03	0.31	T1 vs. T2	15.68	0.07
	T1 vs. T3	31.60	0.001	T1 vs. T3	5.40	0.55
**LVEF**	**cTnI**	**Beta**	**p-value**	**cTnT**	**Beta**	**p-value**
Model 1. Crude association	T1 vs. T2	-0.14	0.92	T1 vs. T2	-2.75	0.06
	T1 vs. T3	-5.18	<0.001	T1 vs. T3	-3.60	0.01
Model 2. + Age & Gender	T1 vs. T2	0.17	0.91	T1 vs. T2	-2.08	0.17
	T1 vs. T3	-4.31	0.004	T1 vs. T3	-2.92	0.05
Model 3. + Diabetes & HT	T1 vs. T2	0.24	0.87	T1 vs. T2	-2.12	0.17
	T1 vs. T3	-4.24	0.007	T1 vs. T3	-2.89	0.06
Model 4. + Dialysis factors	T1 vs. T2	0.03	0.99	T1 vs. T2	-2.60	0.10
	T1 vs. T3	-4.78	0.005	T1 vs. T3	-3.19	0.06

Serum cTnT and cTnI levels were divided into tertiles (T1, T2, and T3). LVMI: Left Ventricular Mass Index; LVEF: Left Ventricular Ejection Fraction; HT: Hypertension.

**Table 4:** Logistic regression for the association between tertiles of serum cardiac troponins and the presence of CAD.

CAD	cTnI	OR	p-value	cTnT	OR	p-value
Model 1. Crude association	T1 vs. T2	2.93	0.03	T1 vs. T2	2.32	0.07
	T1 vs. T3	2.32	0.08	T1 vs. T3	5.13	0.002
Model 2. + Age & Gender	T1 vs. T2	1.97	0.20	T1 vs. T2	1.63	0.33
	T1 vs. T3	1.25	0.68	T1 vs. T3	3.49	0.03
Model 3. + Diabetes & HT	T1 vs. T2	1.47	0.50	T1 vs. T2	1.43	0.49
	T1 vs. T3	0.82	0.73	T1 vs. T3	3.40	0.04
Model 4. + Dialysis factors	T1 vs. T2	2.27	0.19	T1 vs. T2	2.30	0.15
	T1 vs. T3	1.19	0.78	T1 vs. T3	4.70	0.02

Serum cTnT and cTnI levels were divided into tertiles (T1, T2, and T3). CAD; Coronary Artery Disease; HT: Hypertension.

A high serum cTnI (T1 vs. T3) showed a strong crude association with LVMI (Beta 38.72; p = <0.001) and LVEF (Beta -5.18; p = 0.001) in the crude model, which remained robust after fully adjusting for confounders: for LVMI (Beta 31.60; p = <0.001) and for LVEF (Beta -4.78; p = 0.004). An intermediate serum cTnI level (T1 vs. T2) did not show a significant association with LVMI or LVEF in the crude model, or in the fully adjusted models (Table 3). Furthermore, while an intermediate serum cTnI was significantly associated with the presence of CAD in the crude model (OR: 2.93; p = 0.03), this association was no longer statistically significant after adjusting for confounders (Table 4).

A high cTnT showed a significant crude association with LVMI and LVEF (Beta 15.28; p = 0.05 and Beta -3.60; p = 0.01) which was no longer statistically significant after adjusting for confounders (Table 3). A high cTnT was significantly associated with the presence of CAD in the crude model (OR: 5.13; p = 0.002) and this association remained robust after adjusting for confounders (OR 4.70; p = 0.02) (Table 4). An intermediate serum cTnT level showed a significant crude association with LVMI (Beta 23.42; p = 0.004). However, after adjusting for confounders, this association was no longer statistically significant.

## Discussion

This study sought to elucidate the association between cardiac-specific troponins and the presence of CVD in a population of clinically stable dialysis patients. Serum cTnT levels exceeded the 99^th^ percentile in 98% of patients, whereas serum cTnI levels were elevated in only 20%. Patients with the highest tertile of cTnI showed a significantly larger LVMI and lower LVEF compared to patients in the lowest tertile of cTnI after adjusting for confounders. On the other hand, CAD was more prevalent in patients with the highest tertile of serum cTnT compared to patients in the lowest tertile of cTnT. Furthermore, serum cTnT levels were associated with RRF, while cTnI levels were not. These results indicate that, in clinically stable dialysis patients, serum cTnI might be superior to cTnT as a marker for left ventricular functional and structural dysfunction, while cTnT might be better suited to detect CAD than cTnI.

The present study is one of the few studies using high-sensitivity troponin assays to compare the association of cTnT and cTnI with the presence of chronic CVD in patients with ESRD. In a recent paper, Artunc and colleagues elegantly demonstrated that cTnT and cTnI levels might be influenced by dialysis-related factors such as residual diuresis dialysis vintage as well as cardiac variables [8]. In their study an association between cTnT and RRF was found as well, although they used residual urine production rather than the superior urea and creatinine clearances as an indicator of RRF [8, 16]. By investigating a more expansive number of markers for CVD and using a more precise analysis of RRF, we hope to further enhance the current understanding of elevated cardiac troponin values in dialysis patients.

It has been proposed that LVH causes subclinical microvascular heart disease and increased cardiac strain, resulting in leakage of cardiac troponins across the membrane of the hypertrophic cardiomyocyte [20]. Indeed, an association between both troponin isoforms and LVH has been demonstrated in the general population, patients with chronic kidney disease, and patients with ESRD [9, 21–24]. An association between both troponins and a depressed left ventricular systolic function has been shown before as well [8, 23].

In contrast to the previous studies mentioned above however, in the present cohort, serum cTnT levels were not independently associated with LVMI or LVEF. This might be due to the fact that previous studies that reported a positive relationship between cTnT and left ventricular functional and structural dysfunction in dialysis patients used older troponin assays with lower sensitivity and accuracy [23, 25, 26]. Furthermore, most previous studies did not adjust for dialysis parameters or RRF. Moreover, the association found between cTnT and RRF in patients with ESRD might cause accumulation of cTnT independent of cardiac disease, and a loss of sensitivity to detect an elevated LVMI or a depressed LVEF. Based on the present data, we argue that in clinically stable dialysis patients cTnI might reflect left ventricular functional and structural dysfunction better than cTnT.

In the current study, high serum cTnT levels, unlike high serum cTnI levels, were independently associated with CAD after adjusting for confounders. Several previous studies have proposed cTnT as a viable marker of subclinical CAD in patients with ESRD [27–29]. However, the absence of an association between the presence of high cTnI and CAD is intriguing. A possible explanation might be that cTnT levels are elevated on a larger scale following CAD than cTnI. In line with the present data, a recent study on patients with aortic stenosis (but no ESRD) failed to show an association between cTnI and CAD as well [24]. Thus, serum cTnT might be better suited to detect the presence of CAD in clinically stable dialysis patients than serum cTnI.

A final interesting finding in the current study is the negative relationship demonstrated between RRF and cTnT, whereas cTnI showed no such association. While intact free cTnT is too large to be cleared by the kidneys, it has been suggested that circulating cTnT is split into fragments small enough for renal clearance [13]. Indeed, in patients with AMI (but no ESRD) it was shown that serum cTnT levels at 24h after onset of AMI after adjusting for infarct size were dependent of glomerular filtration rate [30]. Furthermore, fragments of cTnT (but not of cTnI) have been identified in urine samples of asymptomatic dialysis patients [31]. Thus, the high circulating levels of cTnT in dialysis patients might be explained by accumulation of cTnT fragments, in addition to cardiac factors. The absence of an association between cTnI and RRF in dialysis patients suggests that cTnI is cleared by another pathway in those patients such as the reticuloendothelial system, or as recently suggested by Gaze et al. by the dialysis procedure itself [11, 14, 32, 33]. The difference in clearance pathway of cTnT and cTnI might explain the relatively weak correlation found between cTnT and cTnI in our cohort of patients (r = 0.72) compared to the much stronger correlation reported in patients with a GFR>60 mL/min per 1.73m^2^ (r≥0.89) [34].

In addition to a difference in clearance pathway, the presence of troponin-specific auto-antibodies and macro-troponins might have influenced detection of serum levels of both troponin isoforms in our cohort as well. Macro-troponins are rare but can result in inappropriately increased troponin levels, due to reduced serum clearance [35, 36]. On the other hand, the release of cardiac troponins can stimulate the production of autoantibodies directed to cardiac-specific troponin, which interferes with troponin detection, and causes false-negative results [37, 38]. Autoantibodies to cTnI have even been linked to progression of heart failure in mice [39]. Little is known about the presence of macro-troponins and troponin autoantibodies in dialysis patients and whether these patients are at greater risk of formation of macro-troponin or autoantibody directed to troponin.

### Clinical Implications

Elevated troponin levels are often attributed to reduced renal clearance in the clinical setting. Although this might be the case for cTnT, our data demonstrates that cTnI levels are not influenced by residual renal function. Over time, this could influence the choice of cardiac troponin assay in patients with kidney disease. Furthermore, cardiac troponins alone or in conjunction with other biomarkers might be used to create a risk stratification score which can help classify dialysis patients in groups with high and low risk for cardiovascular events. More data on this subject is needed, but such risk stratification might even guide clinical decision making in the future. Moreover, it would be interesting to see whether cTnI is superior to cTnT in the detection of acute cardiac ischemia in patients on dialysis therapy.

### Limitations

This study is limited by the relatively small number of patients included, which is partially compensated for by the meticulous screening protocol on CVD applied in the ICD-2 trial. Another limitation of the study is that limited data exists on the in-vitro stability of hs-cTnI and hs-cTnT. While studies have shown that hs-cTnT concentrations are stable after storage times of up to 3 months, no data exists that analyzes cTnT stability after up to 82 months [40, 41]. Data on in vitro stability of cTnI is scarce as well, with a recent study showing a stable hs-cTnI after up to 3 months and a large study showing a stable and cTnI after 3 years [9, 42]. We found no significant difference in cTnI and cTnT levels measured from recently frozen serum compared to older samples. Furthermore due to the nature of the ongoing ICD-2 trial we were unable to report on mortality and morbidity in relation to troponin levels. Unfortunately, the study design cannot distinguish between cause and consequence.

### Conclusion

Both cardiac-specific troponins showed strong univariate associations with cardiovascular disease. However, only a high serum cTnI was independently associated with markers of left ventricular functional and structural dysfunction such as an elevated LVMI or a depressed LVEF. A high serum cTnT on the other hand, was independently associated with the presence of CAD. Furthermore, unlike cTnI, cTnT was associated with residual renal function. Therefore we argue that cTnI might be superior to cTnT as a marker for left ventricular dysfunction while cTnT might be better suited to detect chronic CAD in clinically stable dialysis patients.

## Supporting Information

S1 Dataset Dataset containing information on included patients. (SAV)
